# A Two-Track Model of Huntington’s Disease Pathology: Striatal Atrophy Mediates Maladaptive Immune Dysregulation

**DOI:** 10.3390/ijms27052384

**Published:** 2026-03-04

**Authors:** H. Jeremy Bockholt, Jordan D. Clemsen, Bradley T. Baker, Vince D. Calhoun, Jane S. Paulsen

**Affiliations:** 1 Center for Translational Research in Neuroimaging and Data Science (TReNDS), Georgia State University, Atlanta, GA 30303, USA; jbockholt@gsu.edu (H.J.B.); jclemsen1@student.gsu.edu (J.D.C.); bbaker43@gsu.edu (B.T.B.); vcalhoun@gsu.edu (V.D.C.); 2 School of Electrical and Computer Engineering, Georgia Institute of Technology, Atlanta, GA 30332, USA; 3 Department of Neurology, Emory University, Atlanta, GA 30322, USA; 4 Department of Neurology, University of Wisconsin–Madison, Madison, WI 53706, USA

**Keywords:** Huntington’s disease, proteomics, NULISA, neurodegeneration, striatum, neuroinflammation, biomarkers, neuro-immune axis

## Abstract

Huntington’s disease (HD) is characterized by progressive striatal atrophy and complex proteomic changes in the central nervous system. Using the ultrasensitive Next-Gen Ultra-Sensitive Immunoassay (NULISA) proteomic platform, we analyzed cerebrospinal fluid (CSF) from 88 persons with HD to dissect the biological correlates of gray matter loss. Our findings reveal a distinct “Two-Track” model of pathology. The first track, marked by the axonal damage protein neurofilament light chain (NEFL), showed a strong inverse correlation with putamen volume (Pearson *r* = −0.53, *p* < 0.001), reinforcing its utility as a proxy for structural neurodegeneration. The second track was defined by a positive association between the immune regulator TNFRSF8 (CD30) and putamen volume (Pearson *r* = 0.36, *p* < 0.001), reflecting a decline in active immune-regulatory signaling as striatal atrophy advances. Given its established role in immune modulation, TNFRSF8 was pre-specified for follow-up to further interrogate this neuro-immune axis. Crucially, TNFRSF8 maintained an independent association with striatal volume (Beta = 0.24, *p* = 0.008) even after controlling for NEFL, genetic burden (CAG-Age Product score), and sex. Supplementary analyses confirmed that this structural–immune axis is localized specifically to the striatum—showing no association with generic structural control regions—and is driven by CAG repeat length rather than chronological aging. Furthermore, bidirectional mediation analysis supported an atrophy-driven model, where striatal volume statistically mediates the relationship between genetic burden and downstream immune dysregulation (*p* = 0.010). These results demonstrate that maladaptive immune signaling is a distinct pathological correlate in HD, separable from general cytoskeletal damage. This dual-axis framework warrants evaluation in larger longitudinal and interventional studies to guide future biomarker-driven patient stratification and target engagement.

## 1. Introduction

Huntington’s disease (HD) is a fatal neurodegenerative disorder driven by a Cytosine-Adenine-Guanine (CAG) repeat expansion in the huntingtin (*HTT*) gene [1]. A hallmark of HD pathology is the selective vulnerability and progressive degeneration of striatal medium spiny neurons in the caudate and putamen [2]. Striatal atrophy begins well before the onset of diagnostic motor manifestations, providing a long prodromal window during which neurodegeneration can be tracked using structural magnetic resonance imaging (MRI) [3,4,5,6]. While MRI offers a macroscopic readout of tissue loss, fluid biomarkers are needed to interrogate the molecular processes associated with this degeneration and its modulation over time [7].

Current biomarker research has largely emphasized cytoskeletal markers such as neurofilament light chain (NEFL), which increase in cerebrospinal fluid (CSF) and blood in association with neurodegeneration and track disease intensity and progression in HD. Although NEFL provides a robust gauge of aggregate axonal damage, it is less informative regarding upstream biological processes—such as inflammation and immune dysregulation—that may contribute to or modulate degeneration in HD [8,9]. Advances in high-sensitivity, high-multiplex proteomic technologies now enable improved characterization of low-abundance inflammatory and immune mediators in biofluids. In particular, the Next-Gen Ultra-Sensitive Immunoassay (NULISA) platform supports attomolar-sensitive, multiplexed quantification of large protein panels—including cytokines and other immune-related targets—offering a new window into the immune microenvironment associated with neurodegeneration [10].

While previous research has highlighted broad immune activation and microglial involvement in HD, there is a critical need to evaluate specific immune-regulatory pathways to better understand phenotypic heterogeneity. One such candidate is TNFRSF8 (CD30), a member of the tumor necrosis factor (TNF) receptor superfamily known for its role in immune signaling and regulation. High-sensitivity CSF proteomics now allows us to quantify these low-abundance immune regulators to see whether they index distinct biological tracks of neurodegeneration. In the context of chronic neuroinflammation, we hypothesize that reduced levels of TNFRSF8 in CSF indicate a loss of putative immune-regulatory signaling. This decline would be consistent with an immune-associated signal emerging as striatal atrophy progresses, providing a distinct signal separable from aggregate cytoskeletal injury.

HD progression shows substantial inter-individual variability, and mounting evidence implicates immune dysregulation as one contributor to phenotypic heterogeneity and progression rates. To conceptualize this, we propose a “Two-Track” (or dual-pathway) model of HD. In this framework, Track 1 represents the well-established cascade of structural and cytoskeletal disintegration, robustly captured by markers like NEFL. Track 2 represents a distinct, downstream immune-associated signal, indexed by low-abundance immune-regulatory proteins like TNFRSF8. The practical application of this dual-pathway model is highly relevant for future clinical trials: if these parallel tracks are statistically independent, it implies that interventions targeting structural neurodegeneration may not inherently correct immune dysfunction, necessitating multidimensional biomarker endpoints and combination therapies. In this study, we test whether immune-regulatory signaling measured in CSF explains variance in striatal atrophy that is statistically independent of neuroaxonal damage markers. To ensure that these profiles reflect HD-specific pathogenesis rather than generalized aging or non-specific neurodegeneration, we explicitly evaluate the anatomical specificity of this signal and decouple the effects of chronological age from the underlying genetic mutation. Furthermore, we interrogate the directionality of this neuro-immune axis to determine whether immune dysregulation acts as a primary driver of atrophy or as a secondary, downstream consequence of structural degeneration.

## 2. Results

### 2.1. Validation of Striatal Atrophy

Volumetric screening confirmed significant, disease-specific atrophy in the striatum. The strongest correlation between gray matter volume and disease burden (CAG-Age-Product [CAP] score) was observed in the putamen (Spearman rho = −0.64, *p* < 0.001), followed by the caudate (Spearman rho = −0.44, *p* < 0.001). Preliminary analyses indicated no significant differences between male and female participants in normalized putamen volume (*p* = 0.103), CSF NEFL levels (*p* = 0.820), or CSF TNFRSF8 levels (*p* = 0.700). Measures of global brain volume showed weaker associations, validating the striatum as the primary locus of degeneration in this cohort (Table 1). These patterns are consistent with established longitudinal imaging findings in HD cohorts [4,5,7,11,12].

Table 1 displays the 10 brain regions demonstrating the strongest inverse correlations with disease burden (CAP score). Associations with disease burden were evaluated using Pearson correlation coefficients (R) and their corresponding unadjusted significance levels (*p*-value (CAP)). To adjust for head size, raw regional volumes were divided by total intracranial volume, yielding the standardized values shown as Norm_Stats (SD). For enhanced interpretability of the most highly significant regions, the unadjusted *p*-values are also displayed on a negative logarithmic scale (−log_10_ (*p*)).

To confirm the regional specificity of this immune-structural axis and ensure that it did not merely reflect generalized, age-related neurodegeneration, we evaluated TNFRSF8 against structural control regions. Although TNFRSF8 was robustly correlated with striatal atrophy (both putamen and caudate), it showed no significant association with hippocampal or cerebellar volumes (Table S1). This regional divergence indicates that the TNFRSF8 signature is specifically tied to striatal degeneration rather than global structural decline.

### 2.2. Divergent Proteomic Signatures

We identified two distinct biological signals tracking with striatal atrophy. Screening results are provided in Table S2 (Spearman rho), whereas the region-of-interest associations below are reported as Pearson r.

The Damage Signal (NEFL): Levels of NEFL were strongly negatively correlated with putamen volume (Pearson r = −0.53, *p* < 0.001). As striatal tissue is lost, CSF NEFL concentrations rise, reflecting neuroaxonal injury and release of cytoskeletal debris, consistent with extensive prior work supporting NEFL as a biomarker of neurodegeneration in HD [13,14].

The Immune Signal (TNFRSF8): In a divergent pattern, the immune regulator TNFRSF8 (CD30) showed a positive correlation with putamen volume (Pearson r = 0.36, *p* < 0.001). As the striatum degenerates, levels of this putative immune-regulatory signal increase. TNFRSF8/CD30 is a member of the tumor necrosis factor (TNF) receptor superfamily with immune-regulatory functions, supporting its interpretation as an immune-associated signal in this context [15,16].

**Figure 1:**
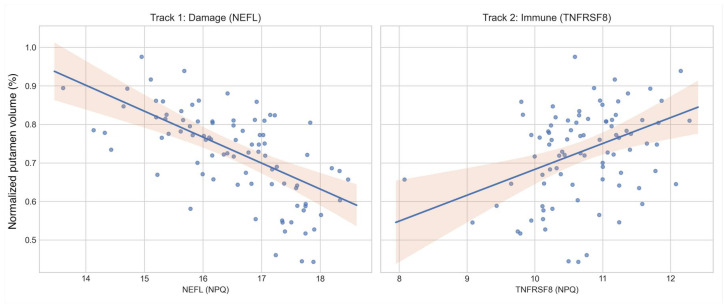
Divergent ‘Two-Track’ signatures of striatal neurodegeneration. Scatterplots show normalized putamen volume (y-axis) versus CSF biofluid markers (x-axis). Biomarker levels are expressed in NULISA Protein Quantification (NPQ) units, a log2-transformed scale. (**Left**) Track 1: Neuroaxonal damage marker NEFL increases as putamen volume declines. (**Right**) Track 2: Immune regulator TNFRSF8 (CD30) decreases as putamen volume declines. Across panels, up indicates larger putamen volume and right indicates higher biomarker level. This divergence is consistent with immune dysregulation and a distinct immune-associated signal.

The correlations differed in direction (Figure 1). Additionally, the difference between dependent correlations was statistically significant (Steiger’s Z = −5.94, *p* < 0.001). Taken together, these patterns support opposing biological behaviors rather than a single underlying factor [17].

### 2.3. Independent Contributions to Atrophy

To determine the optimal baseline for our independence testing, we first performed prespecified disease-burden model comparisons on the complete-case sample (N = 88). The CAP-adjusted model showed the best performance (Adj R^2^ = 0.380; CV RMSE = 0.108), modestly outperforming the age-adjusted model (Adj R^2^ = 0.350; CV RMSE = 0.111) and the no-burden model (Adj R^2^ = 0.355; CV RMSE = 0.110). Based on these results (Table 2), the CAP-adjusted specification served as the base model for subsequent testing.

**Figure 2:**
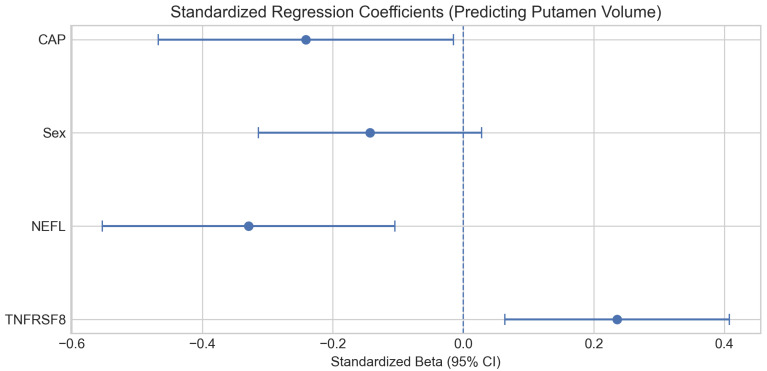
Standardized regression coefficients for the multivariable model predicting normalized putamen volume. Coefficients are adjusted for CAP score and sex, highlighting separable associations for NEFL and TNFRSF8.

To determine whether the immune signal provides unique information beyond this baseline, we performed an independence test using multivariable regression (Figure 2). The base model with CAP score, sex, and NEFL explained 35.6% of the variance in putamen volume (R^2^ = 0.356). Adding TNFRSF8 increased explained variance to 40.9% (R^2^ = 0.409; ΔR^2^ = 0.053), and it significantly improved model fit (*p* = 0.008). TNFRSF8 was statistically significant as an independent predictor (Beta = 0.24, *p* = 0.008) even after controlling for the robust effect of NEFL, cumulative genetic burden (CAP score), and sex. To ensure that this relationship was not confounded by general aging, we ran an additional model decoupling the CAP score into chronological age and CAG repeat length. TNFRSF8 maintained its strong independent association with putamen volume (*p* = 0.008) whereas chronological age did not (*p* = 0.055), confirming that this neuro-immune axis is driven by HD pathogenesis rather than the passage of time (Supplementary Table S3). To mitigate optimism from using the same cohort for screening and modeling, we estimated confidence intervals for Beta via bootstrap resampling (B = 1000). Internal validation confirmed the stability of the TNFRSF8 effect (Beta = 0.24, 95% Bootstrap CI: [0.06, 0.46]) [18].

**Figure 3:**
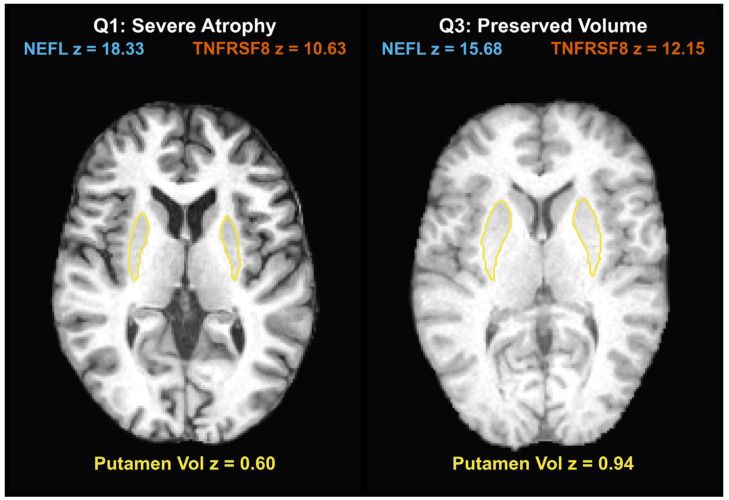
Individual-level exemplars of divergent neuroanatomical and biomarker profiles. Representative axial T1-weighted MRI slices illustrate the relationship between putamen structure and circulating protein biomarkers. Automated segmentations of the putamen are overlaid as yellow contours. Overlay text denotes standardized (Z-scored) values for CSF NEFL (in blue), CSF TNFRSF8 (in red), and total combined putamen volume (in yellow). The left panel demonstrates a profile of severe putamen atrophy (Q1) paired with elevated NEFL and reduced TNFRSF8. This is contrasted with the right panel, which illustrates a clinically divergent phenotype characterized by preserved putamen volume (Q3) and an inverted proteomic signature.

Representative individual-level examples are shown in Figure 3, illustrating two distinct biomarker signatures (high NEFL vs. low TNFRSF8) within the same putamen atrophy quartile.

### 2.4. Mediation Analysis

**Figure 4:**
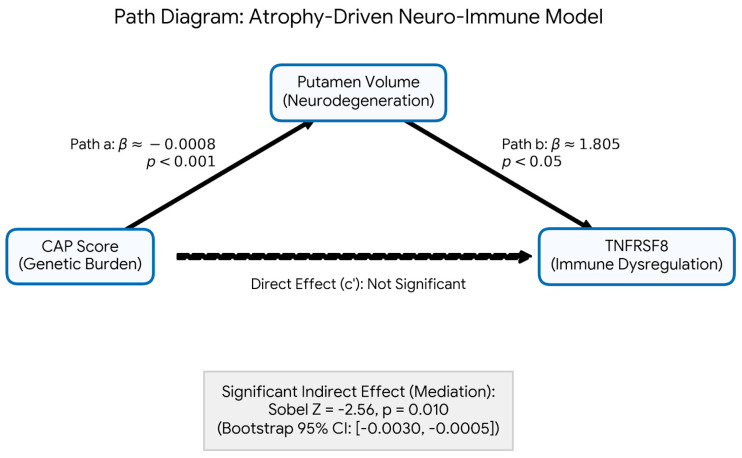
Path diagram of the atrophy-driven neuro-immune model. Bidirectional cross-sectional mediation analysis was used to test the temporal hierarchy of HD pathology. The data strongly support an atrophy-driven directional model, where the effect of genetic burden (CAG-Age Product [CAP] score) on the immune-regulatory marker TNFRSF8 is formally mediated by putamen volume. The indirect effect of this pathway is highly significant (Sobel *Z* = −2.56, *p* = 0.010; Bootstrap 95% CI [−0.0030, −0.0005]). Conversely, the reverse model (testing TNFRSF8 as a mediator of putamen atrophy) was not strongly supported (*p* = 0.106; Supplementary Table S4). These results suggest that central structural neurodegeneration precedes and drives a downstream, maladaptive decline in peripheral immune-regulatory signaling.

To explore the directionality of the neuro-immune axis, we tested alternative causal models. Cross-sectional mediation of CAP effects on putamen volume via TNFRSF8 was not strongly supported (Sobel *p* = 0.106; Supplementary Table S4. Conversely, testing putamen volume as a mediator of CAP effects on TNFRSF8 levels revealed a significant indirect effect (Sobel *Z* = −2.56, *p* = 0.010; bootstrap 95% CI [−0.0030, −0.0005]). This directional asymmetry suggests that structural striatal atrophy mediates the relationship between genetic burden and subsequent immune dysregulation, rather than the reverse (Figure 4).

## 3. Discussion

Our findings propose a “Two-Track” model of HD pathology. Track 1, represented by NEFL, is the familiar signal of structural disintegration—a readout of neuroaxonal injury that is increasingly used in HD biomarker research [13,14]. Track 2, represented by TNFRSF8 (CD30), suggests a distinct, immune-associated signal that declines with advancing striatal degeneration. TNFRSF8/CD30 is a TNF receptor superfamily member with known roles in immune signaling and regulation, and its decline is a hallmark of immune dysregulation, consistent with an interpretation involving reduced immune-regulatory capacity as atrophy progresses [15,16].

This immune interpretation is biologically plausible given a broader literature supporting immune alterations in HD, including immune activation detectable prior to clinical onset, neuroinflammatory pathways implicated in disease progression, and evidence of microglial activation in persons with HD before clinical motor diagnosis [8,19,20,21,22,23]. The positive correlation between TNFRSF8 (CD30) and putamen volume may initially appear counterintuitive. In many neurodegenerative contexts, immune markers rise concurrently with tissue damage as part of a reactive neuroinflammatory response. However, soluble CD30 is primarily shed by actively engaged and proliferating immune cells, particularly activated T-cells. The observation that CD30 levels decline as striatal atrophy advances suggests a longitudinal shift toward a distinct late-stage immune-associated signal. In the context of chronic neuroinflammation, prolonged exposure to disease pathology may lead to profound immune dysregulation, impairing the cells’ ability to maintain active regulatory signaling and blunting the shedding of CD30 into the CSF. Alternatively, this decline may reflect the progressive loss of protective, CD30-expressing regulatory immune cells that normally help maintain neuro-glial homeostasis. Under either mechanism, lower CD30 levels do not indicate an absence of inflammation, but rather a maladaptive failure of active immune-regulatory networks as the disease worsens.

The clinical implication is that targeting the immune system in HD may require a different approach than nonspecific suppression of inflammation. Specifically, if the progressive decline in TNFRSF8/CD30 reflects a distinct, maladaptive immune-associated signal, future HD clinical trials might need to explore immune-modulating therapies designed to restore this specific signaling pathway. Further, utilizing CD30 as an independent biomarker could allow these trials to measure target engagement and immune restoration distinctly from structural cytoskeletal damage. Therefore, future research should evaluate whether preserving or restoring immune-regulatory signaling pathways (e.g., TNF/TNFR superfamily) yields clinical benefit, ideally using designs that can test temporal ordering and within-person coupling [24].

Crucially, our modeling choices and mediation analyses reveal important insights into the fundamental nature of HD progression. The finding that CAP-adjusted models outperformed age-adjusted models reinforces the concept that these structural and immune alterations are driven by the specific toxic genetic burden of the disease—integrating the CAG repeat expansion over time—rather than chronological aging alone [25].

Furthermore, our bidirectional mediation analyses are highly revealing. The data do not support a simple mechanistic chain where genetic burden (CAP) directly causes an immune-associated signal (TNFRSF8 decline), which in turn causes striatal atrophy. Instead, we found significant support for the reverse pathway: putamen volume formally mediates the relationship between CAP score and TNFRSF8 levels. This directional asymmetry provides crucial statistical backing for our hypothesis that TNFRSF8 represents a downstream, late-stage immune-associated signal. It suggests an ‘atrophy-driven’ model of neuro-immune collapse, where the primary genetic insult initiates structural cytoskeletal damage (Track 1), and the accumulating central nervous system burden subsequently drives the dysregulation of peripheral immune-regulatory networks (Track 2). This temporal hierarchy highlights the complexity of the neuro-immune axis in HD, implying that preventing primary structural damage may be a prerequisite for rescuing immune homeostasis.

Because these two tracks provide unique, non-overlapping information, relying solely on single markers like NEFL may obscure critical dimensions of a patient’s disease state. A composite biomarker approach—integrating both neuroaxonal (NEFL) and immune-regulatory (TNFRSF8) readouts—could provide a much higher-resolution, multidimensional picture of HD pathology. Clinically, this dual-axis profiling could prove invaluable for next-generation trial designs, allowing for biomarker-guided patient stratification, prognostic enrichment, and the precise measurement of target engagement for combination therapies that address both neurodegeneration and neuroinflammation [13,14].

### 3.1. Limitations

Despite these findings, this study presents several limitations that may inform future research. First, cross-sectional mediation analysis may be biased in cases where the underlying causal process is dynamic or complex. Second, the comparatively small sample size of 88 persons with HD may limit the statistical power and generalizability of our findings. Third, the current study lacks a control group of gene-negative family members or healthy individuals. While our primary focus was evaluating continuous relationships between biofluid markers and structural atrophy within the HD continuum, the absence of a control cohort precludes direct comparisons to a healthy neuro-immune baseline. However, our supplementary regional specificity and chronological age-decoupling analyses strongly mitigate the concern that these findings merely reflect global aging or generalized neurodegeneration, instead confirming a highly localized, disease-specific striatal axis. Fourth, data regarding concomitant medications were based on self-reported clinical history. The lack of standardized, prospective medication logs or toxicology screening may limit the precision with which we could evaluate the influence of anti-inflammatory or other confounding substances on the CSF proteome.

### 3.2. Future Directions

To directly address these limitations and prospectively validate the disease-specificity observed in our internal structural controls, future work should extend this cross-sectional analysis using larger longitudinal datasets that include gene-negative controls and repeated CSF collection paired to structural MRI. Such designs would enable (i) mixed-effects trajectory models to quantify within-person change in TNFRSF8 and striatal volume, (ii) tests of temporal precedence using time-lagged associations, and (iii) longitudinal mediation or dynamic path models that are not identifiable from cross-sectional data [26,27,28]. Furthermore, expanding this framework into multi-pathway panels will be critical for capturing the full pathophysiological cascade of HD. Future investigations should integrate our findings with parallel markers such as neurofilament heavy chain (NEFH) to complement NEFL as an index of varied axonal pathology [29], myeloperoxidase (MPO) to capture the oxidative stress and innate immune dysregulation characteristic of HD [30,31], and ubiquitin C-terminal hydrolase L1 (UCHL1). As a core component of the ubiquitin-proteasome system, UCHL1 serves not only as a direct measure of neuronal somatic injury but also reflects the breakdown of proteostasis central to mutant huntingtin aggregation [32,33]. With larger controlled longitudinal samples, these expanded multi-marker approaches could be definitively evaluated for prognostic enrichment and sensitivity to therapeutic target engagement in interventional studies.

## 4. Materials and Methods

### 4.1. Participants

We analyzed data from 88 people with HD (CAG repeats ≥36) spanning the stages before clinical motor diagnosis and the early stages after clinical motor diagnosis [34,35]. To minimize confounding effects on biofluid immune markers, patients taking potent immunomodulatory therapies were excluded from the study. Concomitant medications were screened for use of common anti-inflammatories, including aspirin, ibuprofen, and other non-steroidal anti-inflammatory drugs (NSAID). Demographics and clinical characteristics are summarized in Table 3. The cohort had a mean age of 39.0 years (SD = 11.8) and a mean CAP score of 336.5 (SD = 92.3) [25,36].

### 4.2. Neuroimaging

T1-weighted MRI scans were acquired on GE (GE HealthCare Technologies Inc. in Chicago, IL, USA) 3T scanners using harmonized protocols across sites and processed with FastSurfer (German Center for Neurodegenerative Diseases [DZNE], Bonn, Germany) to generate automated segmentations of subcortical structures [37]. Caudate and putamen volumes were normalized to intracranial volume (ICV) using the ratio method (Volume/ICV). To evaluate regional specificity, bilateral volumes of the hippocampus and cerebellum (including both cerebellar cortex and white matter) were also extracted and similarly normalized to ICV to serve as structural control regions. For the present analysis, which required paired CSF and MRI, we restricted the sample to participants with both structural MRI and CSF available from the University of Iowa and the University of Wisconsin–Madison.

### 4.3. CSF Collection and Handling

CSF samples included in this analysis were collected at the University of Iowa and the University of Wisconsin–Madison under standardized protocols, processed according to study procedures, and stored until assay. Samples were shipped to the BioSpecimen Exchange for Neurological Disorders (BioSEND) repository at Indiana University for centralized handling, where they were blinded and aliquoted before shipment to the University of Wisconsin Alzheimer’s Disease Research Center (ADRC) Biomarker Lab for analysis [38]. Following completion of laboratory assays, results were unblinded, enabling linkage of CSF measures to imaging-derived outcomes for the present conjoined analyses.

### 4.4. Proteomics

Proteomic profiling was performed using the NULISAseq Central Nervous System (CNS) Disease Panel 120 and Inflammation Panel (NIF) 250 (Alamar Biosciences, Fremont, CA, USA), targeting a combined total of approximately 370 proteins [10,39,40]. Data were normalized using internal and inter-plate controls and log2-transformed to the NULISA Protein Quantification (NPQ) scale [10]. Values below the limit of detection (LOD) were retained (as NPQ values) consistent with manufacturer recommendations, and low-detectability analytes were filtered prior to screening, yielding 268 proteins for downstream analysis [10].

### 4.5. Statistical Analysis and Biomarker Screening

Sex was included a priori as a biological covariate in volumetric models. Preliminary group comparisons by sex were evaluated using Welch’s *t*-tests. Other potential covariates (education, study site/scanner, race/ethnicity, and clinical severity measures such as total motor score) were not included in the primary model because they can be sparse/collinear in this sample and, for some measures, may be downstream of neurodegeneration. These were reserved for sensitivity analyses.

Screening procedure: To identify candidate biomarkers of striatal neurodegeneration, we screened the 268 retained proteins by computing Spearman’s rank correlations between protein abundance and normalized putamen volume. *p*-values were adjusted using the Benjamini–Hochberg false discovery rate (FDR) procedure [41]. Significance was defined as q < 0.05, and analytes meeting a suggestive threshold (q < 0.10) were advanced for multivariable characterization. TNFRSF8 was pre-specified for follow-up given its immune-regulatory role and interpretability as a distinct immune-associated signal in the context of broader immune dysregulation; in this screen, it met the suggestive FDR threshold (q = 0.059).

Multivariable modeling: Analytes meeting the suggestive FDR threshold (q < 0.10) were entered into multivariable linear regression models. In addition, TNFRSF8 was included a priori as a pre-specified immune-regulatory target given its interpretability in the context of immune dysregulation. All continuous predictors (CAP score, NEFL, and candidate immune markers) were Z-score standardized prior to modeling to enable direct comparison of standardized regression coefficients (Beta). The primary model was specified as follows: Putamen_Volume ~ CAP + Sex + NEFL + TNFRSF8. While the screening step used Spearman correlations to reduce sensitivity to outliers, region-of-interest matrices and multivariable models used Pearson correlations and ordinary least squares (OLS) regression on standardized data to satisfy linear modeling assumptions.

Disease-burden model comparison: We compared three prespecified models on a common complete-case sample (N = 88): (i) no disease-burden covariate, (ii) age-adjusted, and (iii) CAP-adjusted. Models were compared using adjusted R2, AIC/BIC, and k-fold cross-validated RMSE/MAE (k = 10 when available). CAP was retained as the primary disease-burden covariate because it provided superior fit and/or lower cross-validated error relative to age.

Regional Specificity and Age-Decoupling Analyses: To ensure the identified neuro-immune associations reflected HD-specific pathogenesis rather than generalized aging or non-specific neurodegeneration, two pre-specified supplementary analyses were conducted. First, regional specificity was evaluated by calculating Pearson correlations between TNFRSF8 and the ICV-normalized volumes of structural control regions (hippocampus and cerebellum). Second, to mathematically decouple the effects of chronological aging from genetic burden, an additional multivariable OLS regression model was conducted. In this model, the composite CAP score was separated into its constituent variables—chronological age and CAG repeat length—to determine whether TNFRSF8 maintained a significant association with putamen volume independent of chronological age.

Model diagnostics: Multicollinearity was assessed using the variance inflation factor (VIF) [42]; all predictors had VIF < 3 (max VIF = 1.80). Residuals were evaluated for normality (Shapiro–Wilk) and homoscedasticity (Breusch–Pagan) [43,44]. Influential observations were assessed using Cook’s distance (threshold 4/N) [45].

Sensitivity analyses were performed to evaluate the potential influence of concomitant anti-inflammatory medications. No significant associations were observed between the use of aspirin or other NSAID and CSF TNFRSF8 levels. It is, however, noted that the number of patients taking these medications was minimal in the current cohort.

Validation and limitations: To mitigate optimism from using the same cohort for screening and modeling, confidence intervals for regression coefficients were estimated via bootstrap resampling (B = 1000) [18]. Mediation analysis was conducted to assess statistical mediation, evaluating whether TNFRSF8 mediated the association between CAP score and putamen volume [46,47]. All results are presented as exploratory pending external replication.

### 4.6. Generative Artificial Intelligence

The study employed a paper-as-code workflow, in which pre-specified analysis scripts automatically generate tables, figures, and corresponding manuscript text directly from the finalized statistical outputs. This approach ensures internal consistency, reproducibility, and traceability between the analysis code and the reported results.

Any automated text generation was deterministic and rule-based, reflecting fixed templates populated by computed results, rather than probabilistic or generative modeling. No large language model or generative AI system was used to infer, summarize, or interpret findings beyond formatting and structured insertion of results into manuscript templates. All analytical decisions, statistical modeling choices, and scientific interpretations were defined a priori by the authors and verified manually prior to submission.

## 5. Conclusions

In summary, our study finds that altered immune signaling is a distinct pathological correlate in Huntington’s disease, separable from general cytoskeletal damage and independent of chronological aging. Our findings support a “Two-Track” model of HD pathology: Track 1, represented by NEFL, tracks structural disintegration while Track 2, represented by TNFRSF8 (CD30), reflects a parallel immune-associated signal that declines with advancing striatal degeneration. This separation highlights a specific, maladaptive pattern of immune dysregulation that occurs alongside physical atrophy. Ultimately, this study enhances our understanding of the multi-dimensional biological processes that drive HD progression. By extending this framework into larger, longitudinal cohorts, the field can further validate comprehensive multi-marker panels. Such approaches hold significant promise for prognostic enrichment, patient stratification, and the precise measurement of target engagement in future immune-modulating clinical trials for persons with HD.

## Figures and Tables

**Table 1:** Volumetric Screening (Top 10 Regions).

Region	R_CAP	P_CAP	Norm_Stats	−log_10_ (*p*)
Putamen	−0.64	1.98 × 10^−11^	0.73 (0.13)	10.70
Total Brain Volume	−0.54	5.64 × 10^−8^	197.94 (0.91)	7.25
Nucleus Accumbens	−0.48	1.70 × 10^−6^	0.10 (0.02)	5.77
Forebrain	−0.46	7.18 × 10^−6^	173.48 (2.59)	5.14
Caudate	−0.44	1.41 × 10^−5^	0.53 (0.11)	4.85
Pallidum	−0.44	1.88 × 10^−5^	0.29 (0.05)	4.73
Subcortical Gray Matter	−0.42	3.83 × 10^−5^	4.74 (0.47)	4.42
Caudal Middle Frontal Gyrus Right	−0.38	2.76 × 10^−4^	0.49 (0.09)	3.56
Postcentral Gyrus Left	−0.34	0.001	0.89 (0.13)	2.92
Precentral Gyrus Right	−0.33	0.002	1.01 (0.14)	2.75

**Abbreviations:** CAP = CAG-Age-Product; SD = Standard Deviation.

**Table 2:** Disease-burden covariate model comparison for putamen prediction.

Model	N_Complete_Case	ADJ_R2	AIC	BIC	CV_Folds	CV_RMSE	CV_MAE
No burden (no Age/CAP)	88	0.355	−137.9	−128.0	10	0.110	0.076
Age-adjusted	88	0.350	−136.2	−123.8	10	0.111	0.078
CAP-adjusted	88	0.380	−140.5	−128.1	10	0.108	0.075

**Abbreviations:** ADJ_R2 = Adjusted R-squared; AIC = Akaike Information Criterion; BIC = Bayesian Information Criterion; CAP = CAG-Age-Product; CV = Cross-Validated; RMSE = Root Mean Square Error; MAE = Mean Absolute Error.

**Table 3:** Participant demographics.

**Characteristic**	**Cohort (N = 88)**
Age, years, mean (SD)	39.01 (11.83)
Education, years, mean (SD)	15.29 (2.16)
CAP score, mean (SD)	336.48 (92.25)
CAG Repeats, mean (SD)	42.74 (2.93)
**Sex, n (%)**	
Female	58 (65.9%)
Male	30 (34.1%)
**Race, n (%)**	
White	87 (98.9%)
Other/Not Reported	1 (1.1%)
**Ethnicity, n (%)**	
Non-Hispanic	84 (95.5%)
Hispanic	3 (3.4%)
Other/Not Reported	1 (1.1%)

**Abbreviations:** CAP = CAG-Age-Product; CAG = Cytosine-Adenine-Guanine.

## Data Availability

The raw data supporting the conclusions of this article will be made available by the authors on request, in accordance with the data sharing policies of the Predict-HD and PREVENT-HD studies.

## Supplementary Materials

The following supporting information can be downloaded at: https://www.mdpi.com/article/10.3390/ijms27052384/s1.

## Abbreviations

The following abbreviations are used in this manuscript:

ADRC	Alzheimer’s Disease Research Center
BioSEND	BioSpecimen Exchange for Neurological Disorders
CAG	Cytosine-Adenine-Guanine
CAP	CAG-Age-Product
CNS	Central nervous system
CSF	Cerebrospinal fluid
FDR	False discovery rate
HD	Huntington’s Disease
HTT	Huntingtin gene
ICV	Intracranial volume
LOD	Limit of detection
MPO	Myeloperoxidase
MRI	Magnetic resonance imaging
NEFH	Neurofilament heavy chain
NEFL	Neurofilament light chain
NPQ	NULISA Protein Quantification
NSAID	non-steroidal anti-inflammatory drugs
NULISA	Next-Gen Ultra-Sensitive Immunoassay
OLS	Ordinary least squares
TNF	Tumor necrosis factor
UCHL1	Ubiquitin C-terminal hydrolase L1
VIF	Variance inflation factor
