# Supplementary material for: A Two-Track Model of Huntington’s Disease Pathology: Striatal Atrophy Mediates Maladaptive Immune Dysregulation
Source: Int J Mol Sci. 2026 Mar 4;27(5):2384. doi: 10.3390/ijms27052384 (PMC12985888; doi:10.3390/ijms27052384)
Supplement: Supplementary file 1 [file ijms-27-02384-s001.zip › ijms-4140054-supplementary.pdf]

**Table S1: Regional specificity of the TNFRSF8-structural axis.**

| Brain Region (ETIV-Normalized) | Pearson r | p-value | Significance |
|---|---|---|---|
| **Caudate** | **0.396** | **0.0001** | *** |
| **Putamen** | **0.356** | **0.0007** | *** |
| Hippocampus | 0.051 | 0.636 | ns |
| Cerebellum | -0.119 | 0.272 | ns |

**Table S1 Legend:** To determine whether the association between CSF TNFRSF8 and striatal volume reflects Huntington's disease-specific pathology versus global age-related neurodegeneration, correlations were assessed across target and structural control regions. Regional volumes were normalized by Estimated Total Intracranial Volume (ETIV). TNFRSF8 was highly correlated with both components of the striatum (the putamen and caudate, representing the primary sites of HD pathology). In contrast, it showed no significant correlation with normalized hippocampal (a region highly susceptible to general aging) or cerebellar volumes. This regional divergence supports a tightly coupled, localized neuro-immune axis.

**Table S2. Bivariate Associations with Normalized Putamen Volume**

| Analyte | Correlation (r) | p-value | FDR (q-value) | $-\log_{10}(p)$ | $-\log_{10}(FDR)$ |
|---|---|---|---|---|---|
| **NEFL** | -0.65 | $6.30 \times 10^{-12}$ | **$1.66 \times 10^{-9}$** | 11.20 | 8.78 |
| **NEFH** | -0.44 | $2.03 \times 10^{-5}$ | **0.0027** | 4.69 | 2.57 |
| **MPO** | 0.36 | $5.31 \times 10^{-4}$ | **0.0467** | 3.27 | 1.33 |
| *TNFRSF8* | 0.35 | $8.96 \times 10^{-4}$ | *0.0588* | 3.05 | 1.23 |
| UCHL1 | -0.34 | 0.001 | 0.0588 | 2.95 | 1.23 |
| CCL1 | -0.30 | 0.004 | 0.1922 | 2.36 | 0.72 |
| GDI1 | -0.28 | 0.008 | 0.2895 | 2.09 | 0.54 |
| PGK1 | -0.28 | 0.009 | 0.2895 | 2.04 | 0.54 |
| IL2RB | -0.27 | 0.010 | 0.2895 | 2.01 | 0.54 |
| FLT4 | 0.27 | 0.012 | 0.3042 | 1.94 | 0.52 |
| UBB | 0.26 | 0.013 | 0.3161 | 1.88 | 0.50 |
| PRDX6 | -0.26 | 0.016 | 0.3480 | 1.80 | 0.46 |
| PDGFB | -0.25 | 0.020 | 0.4036 | 1.70 | 0.39 |
| CXCL2 | -0.24 | 0.023 | 0.4376 | 1.63 | 0.36 |
| CD80 | -0.23 | 0.029 | 0.4886 | 1.54 | 0.31 |
| IL1RN | -0.23 | 0.030 | 0.4886 | 1.53 | 0.31 |
| FGF19 | 0.22 | 0.037 | 0.5455 | 1.44 | 0.26 |
| CSF1 | -0.22 | 0.037 | 0.5455 | 1.43 | 0.26 |
| MERTK | -0.22 | 0.039 | 0.5455 | 1.41 | 0.26 |
| IL36G | -0.22 | 0.041 | 0.5470 | 1.38 | 0.26 |

**Table S3: Multiple regression analysis decoupling chronological age and genetic burden**

| **Predictor** | **Coefficient (β)** | **Std. Error** | **t-value** | **p-value** | **95% CI** |
|---|---|---|---|---|---|
| **TNFRSF8** | 0.047 | 0.017 | 2.713 | **0.008**** | [0.013, 0.082] |
| **NEFL** | -0.038 | 0.016 | -2.402 | **0.019*** | [-0.069, -0.006] |
| **CAG Repeat Length** | -0.013 | 0.006 | -2.139 | **0.035*** | [-0.026, -0.001] |
| **Chronological Age** | -0.003 | 0.002 | -1.948 | 0.055 | [-0.006, <0.001] |
| **Sex (Male)** | -0.040 | 0.024 | -1.641 | 0.105 | [-0.088, 0.008] |
| *(Note: Model Intercept = 1.546, p < 0.001)* | | | | | |

**Table S3 Legend:** To ensure that the association between CSF TNFRSF8 and putamen volume was not driven by generalized chronological aging, a supplementary multiple regression model was performed. The composite genetic burden score (CAG-Age Product) was decoupled into its constituent parts: Chronological Age and CAG repeat length. Even when adjusting for generic neuroaxonal damage (NEFL) and sex, TNFRSF8 maintained a highly significant, independent association with putamen volume ($p = 0.008$). Furthermore, the genetic driver of Huntington's disease (CAG repeat length) remained a significant predictor ($p = 0.035$) whereas chronological age lost statistical significance ($p = 0.055$). This demonstrates that the structural-immune axis is specific to HD pathogenesis rather than the passage of time.

**Table S4: Bidirectional cross-sectional mediation analysis of the neuro-immune axis.**

| Model (Direction) | N | a (CAP → Mediator) | b (Mediator → Outcome \| CAP) | indirect (a·b) | boot_B | boot CI (low, high) | Sobel Z | Sobel p |
|---|---|---|---|---|---|---|---|---|
| **Atrophy-Driven (Dir 2)**: CAP → **Putamen** → TNFRSF8 | 88 | -7.62e-04 | 1.81 | -1.38e-03 | 5000 | (-2.98e-03, -5.44e-04) | -2.56 | **0.0105** |
| **Immune-Driven (Dir 1)**: CAP → **TNFRSF8** → Putamen | 88 | -1.60e-03 | 4.89e-02 | -7.80e-05 | 5000 | (-1.99e-04, -8.96e-07) | -1.62 | 0.106 |

**Table S4 Legend:** Bidirectional mediation models evaluating the relationship between genetic burden, putamen volume, and immune dysregulation. Path *a* represents the unstandardized beta coefficient for the effect of the independent variable (X) on the mediator (M). Path *b* represents the effect of the mediator on the dependent variable (Y), controlling for X. The indirect effect (*ab*) was tested for significance using both the Sobel test and 5,000 bootstrap resamples to generate 95% confidence intervals. **Model 1** tested whether immune dysregulation (TNFRSF8) mediates structural atrophy (Putamen), which was not strongly supported ($p$ = 0.106). **Model 2** tested whether structural atrophy mediates downstream immune dysregulation, revealing a highly significant indirect effect ($p$ = 0.010). *Abbreviations: CAP, CAG-Age Product score.*
